# Syndrome of Subjective Doubles: Delusions of Identity and Duplication

**DOI:** 10.7759/cureus.81975

**Published:** 2025-04-09

**Authors:** Nayeli Gonzalez, Emmanuel Cabello, Richard M Dreize

**Affiliations:** 1 Psychiatry, Ross University School of Medicine, Miami, USA; 2 Psychiatry, Jackson Behavioral Health Hospital, Miami, USA

**Keywords:** capgras syndrome, delusional misidentification syndromes, delusions of identity, dms, reverse capgras syndrome, syndrome of subjective doubles

## Abstract

The syndrome of subjective doubles is part of the delusional misidentification syndrome (DMS). In this condition, the patient believes that duplicates of themselves or others exist, often with distinct intentions or characteristics. It is associated with psychiatric and neurological disorders. In this report, we describe the case of a 23-year-old patient experiencing the syndrome of subjective doubles. He endorsed psychosis, and the delusion centered on the belief that a musician on social media is identical to him. His delusion eventually improved with medication management and supportive psychotherapy. This case is presented as an important addition to the literature, aiming to raise awareness of a rare disorder with a poorly understood etiology that resulted in a positive outcome.

## Introduction

The syndrome of subjective doubles falls under delusional misidentification syndromes (DMSs). Individuals with this condition believe that duplicates of themselves, or sometimes of others, exist, typically possessing separate intentions or traits [1]. The syndrome of subjective doubles was first described in 1978 by the Greek psychiatrist George N. Christodoulou [1]. In the American Journal of Psychiatry, he reported the case of an 18-year-old female who became convinced that a neighbor had acquired physical characteristics identical to her own [1]. Cases of the syndrome of subjective doubles are not well documented due to its rarity and being a subtype of delusional misidentification syndromes (DMSs). The International Classification of Diseases, Eleventh Revision (ICD-11) recognizes DMSs, falling under the category of misidentification delusion with a specific diagnostic code MB26.0B, while the Diagnostic and Statistical Manual of Mental Disorders, Fifth Edition (DSM-5) does not have a specific code [2]. Cases often occur in individuals with underlying psychiatric conditions such as schizophrenia, bipolar disorder, traumatic brain injuries, and dementia [3,4]. This paper aims to report a case of a young male patient with a syndrome of subjective doubles with unclear etiology and provide valuable insight into this rare condition, potentially improving diagnostic and treatment strategies. 

## Case presentation

This is a case of a 23-year-old male patient with no past psychiatric history and significant substance use, particularly daily marijuana use through smoking and occasional use of mushrooms and lysergic acid diethylamide (LSD). The patient presented to the psychiatric emergency room voluntarily due to being unsure whether he was the real version of himself or “dead” after encountering a musician on social media whom he believed to be identical to him. He states this had been going on for a "few years" and decided to seek help since he felt increasingly distressed and confused by the persistent belief that there was a duplicate of himself. On initial evaluation, the patient was well-groomed and had a calm and congruent affect, with speech characterized by low volume and prolonged latency. The father stated he had become increasingly withdrawn at home and had quit his job as a pool attendant two months ago, which the patient stated was due to feeling sad that his friend had died of “tuberculosis.” The patient reported a history of insomnia, experiencing multiple episodes of sleep deprivation lasting two to three days, with one episode extending up to a week. The patient stated that he had never received treatment for insomnia and denied experiencing insomnia at the time of evaluation. The patient has no past medical history or family history of psychiatric illness and currently lives with his father. The father stated in the ED that the patient had been abstinent from marijuana, LSD, and mushrooms for about a month. The patient was vague about the timing of his last drug use but did acknowledge daily marijuana use and had never received treatment for substance abuse in the past. The patient's vital signs, including blood pressure (BP), pulse rate (PR), respiratory rate (RR), temperature, and peripheral oxygen saturation (SpO2), were within normal limits. General and systemic examinations showed no significant abnormalities. 

The mental status examination showed a patient oriented to time, place, and person, with soft speech and latency of responses. He exhibited a depressed mood and described feeling “numb,” with a congruent affect. His thought process was disorganized and tangential, dominated by a fixed and persistent delusional belief in the existence of his double for an unknown duration, a musician on social media he had seen eight years ago, whom he believed was his duplicate. 

The patient also reported auditory hallucinations, describing “an external voice” making non-command yet derogatory comments. He denied thought insertion and ideas of reference but admitted to thought broadcasting. The patient exhibited paranoia, believing he was being observed, and stated that he felt like the main character of the film, "The Truman Show." The patient was unable to specify the exact duration of these symptoms, stating only that they had been present for "years." 

The patient denied any suicidal or homicidal ideation but expressed significant distress related to the delusion, stating, 'I am a musician too; the only way I can become famous is if I look different from him, and I need to change my face.' He reported playing the guitar as a hobby and expressed a desire to become a renowned musician in the future.

Baseline investigations showed no abnormalities. Routine blood tests, Table 1, include complete blood count (CBC), renal panel, liver function test (LFT), thyroid function test (TFT), fasting blood sugar (FBS), glycated hemoglobin (HbA1c), B12, and folate, and were all normal. Serological tests for human immunodeficiency virus (HIV), hepatitis C antibody, and syphilis IgG/IgM were non-reactive. Urine toxicology results were positive only for cannabis. 

**Table 1:** Comprehensive laboratory results with reference ranges THC: tetrahydrocannabinol; PCR: polymerase chain reaction; SGPT: serum glutamic-pyruvic transaminase; SGOT: serum glutamic-oxaloacetic transaminase; CKD-EPI: chronic kidney disease epidemiology collaboration

Test Name	Result	Reference Range
Complete Blood Count (CBC)		
Red Blood Cell (RBC) Count	5.01 x10⁶ cells/μL	4.5–5.9 x10⁶ cells/μL
Hematocrit (Hct)	44.3%	41%–50%
Mean Corpuscular Hemoglobin (MCH)	28.9 pg	27–31 pg
Mean Corpuscular Hemoglobin Concentration (MCHC)	32.7 g/dL	32–36 g/dL
Mean Platelet Volume (MPV)	10.6 fL	7.5–11.5 fL
Red Cell Distribution Width (RDW-CV)	12.1%	11.5%–14.5%
Neutrophil Count (Absolute)	3.8 x10³/μL	1.5–8.0 x10³/μL
Neutrophils (%)	52.4%	40%–60%
Lymphocytes (Absolute)	2.9 x10³/μL	1.0–4.0 x10³/μL
Lymphocytes (%)	39.6%	20%–40%
Monocyte (%)	6.3%	2%–8%
Eosinophils (%)	0.7%	1%–4%
Basophils (%)	0.7%	0.5%–1%
Immature Granulocyte (%)	0.3%	0%–0.5%
Renal Panel		
Estimated Glomerular Filtration Rate (eGFR) CKD-EPI	>90 mL/min/1.73m²	-
Estimated Creatinine Clearance	178.29 mL/min	90–140 mL/min
Chloride	100 mmol/L	98–106 mmol/L
Calcium	10.2 mg/dL	8.5–10.2 mg/dL
Phosphorus	4.3 mg/dL	2.5–4.5 mg/dL
Liver Function Test (LFT)		
Albumin	4.8 g/dL	3.4–5.4 g/dL
Total Protein	7.5 g/dL	6.0–8.3 g/dL
Alkaline Phosphatase (ALP)	41 unit/L	44–147 unit/L
Alanine Aminotransferase (ALT/SGPT)	13 unit/L	7–56 unit/L
Aspartate Aminotransferase (AST/SGOT)	27 unit/L	10–40 unit/L
Gamma-glutamyl Transferase (GGTP)	14 unit/L	9–48 unit/L
Total Bilirubin	1.2 mg/dL	0.1–1.2 mg/dL
Thyroid Function Test (TFT)		
Thyroid-Stimulating Hormone (TSH)	0.753 mIU/mL	0.4–4.0 mIU/mL
Blood Sugar Tests		
Fasting Blood Sugar (FBS)	93 mg/dL	70–100 mg/dL
Glycated Hemoglobin (HbA1c)	5.4%	4%–5.6%
Vitamin Levels		
Vitamin B12	470 pg/mL	200–900 pg/mL
Folate	7.7 ng/mL	2.7–17.0 ng/mL
Serological Tests		
HIV Antibody/Antigen Test	Negative	-
HIV DNA PCR	Not Detected	-
Hepatitis C Antibody	0.01 s/co ratio (Negative)	-
Syphilis IgG/IgM	Non-reactive	-
Drug Screen		
Cannabis (THC)	Presumptive Positive	-

The differential diagnoses initially considered included Capgras syndrome, reverse Capgras syndrome, and syndrome of subjective doubles. Capgras syndrome was unlikely since he did not believe that someone significant in his life was replaced by an identical impostor [4]. Reverse Capgras syndrome was also ruled out since the patient did not believe he was replaced by an impostor but rather that there was someone identical to him, which was causing him significant distress [5]. Although Capgras syndrome and its variant, reverse Capgras, can co-occur with the syndrome of subjective doubles, he did not appear to be exhibiting either [6]. Therefore, the most appropriate diagnosis for the patient was a syndrome of subjective doubles with a possible onset of schizophrenia. The diagnosis was established based on the DSM-5 criteria for schizophrenia, considering the patient's reported symptoms [7].

The patient was admitted inpatient in the behavioral health unit and started on 5 mg of olanzapine nightly and attended group therapy occasionally. The patient slowly started showing improvement, becoming less preoccupied with his double and experiencing a reduction in the frequency and intensity of auditory hallucinations. During the five days of hospitalization, he was asked how strongly he believed that the musician on social media was his double. Initially, he adamantly believed that the musician was his double despite the clinical team observing no physical resemblance. As the days progressed, he remained concerned about the double but admitted to feeling less distressed and acknowledged that the medication was helping him. By his last day in the hospital, the auditory hallucinations involving derogatory remarks had completely subsided, and his delusion of having a double had significantly diminished. The patient now denied experiencing thought broadcasting or paranoia about being watched. He was discharged with an appointment for an outpatient psychiatrist and a prescription for 5 mg of olanzapine at bedtime, with the goal of titration as necessary on subsequent follow-up. The patient was lost to follow-up, making it impossible to determine whether the symptoms fully subsided or if there was an overall improvement in his quality of life with the medication.

## Discussion

The syndrome of subjective doubles (SSD) is a rare subtype of delusional misidentification syndromes (DMSs) that is grossly underreported, presenting unique challenges in both diagnosis and treatment. According to existing literature, there is no established first-line treatment for SSD, though evidence suggests that antipsychotics such as olanzapine, trifluoperazine, clorazepate, and pimozide have shown positive outcomes [5]. Given that DMS can manifest in psychiatric conditions such as schizophrenia, schizoaffective disorder, or bipolar disorder, the use of antipsychotic medications and mood stabilizers appears to be the most appropriate treatment approach [5,8]. The positive response to olanzapine in our patient's case adds to the growing body of literature supporting its effectiveness in managing conditions like SSD [9,10,11]. 

In a case study, the patient, similar to ours, was suffering from the subtype, syndrome of subjective doubles and paranoid ideation. The patient was in his 30s and believed that a second version of himself had duplicated his identity three years earlier after a friend performed a witchcraft ritual on him. He also exhibited magical thinking, persecutory and self-referential delusions, but did not respond to internal stimuli. Similar to our case, the patient had no family history of mental illness but reported chronic cannabis use over a 10-year period, with urine toxicology testing positive for cannabis. Treatment was initiated with paliperidone 6 mg PO, lorazepam 1 mg PO, and folic acid 5 mg PO. After five days, paliperidone was increased to 9 mg PO daily. He also received cognitive behavioral therapy, motivational interviewing, and counseling on drug use. The patient reached full remission after seven days with no remaining signs of delusions or perception disturbances. He was discharged with paliperidone 9 mg PO and folic acid 5 mg PO, with a plan to follow up [12].

A similar case study on DMS described a 22-year-old patient suffering from Capgras syndrome, delusion of subjective doubles, and reverse intermetamorphosis. The patient believed that his parents had been replaced by impostors, that he had a “carbon copy” of himself, and that he was his cousin while the “real” version of himself had been kidnapped. Like our patient, he had no family history of mental illness and was diagnosed with schizophrenia due to persistently active delusions extending beyond a month. Treatment with olanzapine, starting at 5 mg and titrated up to 10 mg, led to significant symptom improvement. The patient was ultimately stabilized on 15 mg of olanzapine on follow-up sessions [9]. 

In another case study on DMS, specifically the subtype Capgras syndrome, olanzapine also showed the ability to greatly improve psychotic symptoms, just like in our patient. In this case, a 39-year-old patient with schizophrenia exhibited paranoia, visual hallucinations, and the belief that his father was an impostor. This patient, like ours, was a chronic cannabis user. He showed major improvement when treated with olanzapine, which was titrated over three days to 15 mg daily. Following treatment, his delusions diminished, and his speech became more linear and logical [11].

An additional case study on the subtype, reverse Capgras syndrome, detailed a 25-year-old patient who believed she was the convicted sex offender Jeffrey Epstein. The patient experienced paranoid delusions and auditory hallucinations involving derogatory remarks from Epstein. Her mother suspected that the patient had been using marijuana with her partner before symptom onset, similar to our patient’s history. She was started on 5 mg of olanzapine and gradually titrated to 25 mg, with steady improvement throughout a four-week hospitalization. By discharge, her delusion had completely resolved, and she reported feeling like her “true self” again [10].

These cases collectively illustrate the ability of olanzapine to improve delusional misidentification syndromes (DMS), and the dosage varies depending on individual patient factors. Notably, three out of the four cases involved chronic cannabis use, as seen in our patient. This finding highlights the need for further research on how marijuana use may exacerbate or trigger psychotic symptoms, including delusions, particularly in individuals with underlying vulnerabilities like a history of psychiatric disorders or brain injuries [4,13,14]. Recent literature has already been reporting on cannabis being a likely trigger for DMS, although more research is needed to identify whether it is an inciting factor and understand the neurobiological pathways involved [13]. 

Given that the syndrome of subjective doubles (SSD) and other delusional misidentification syndromes (DMSs) are highly underreported and rare, this case sheds light on the fact that further studies are needed in order to establish clear diagnostic criteria, understand the pathophysiology and the role of substance use, and set guidelines for the first-line treatment options for DMS. 

## Conclusions

Syndrome of subjective doubles (SSD) is an extremely rare disturbance of self-identity, where individuals perceive a "double" or alternate version of themselves that may act independently or embody distinct aspects of their personality. In this case, no specific trigger was identified, demonstrating the complexity of this syndrome. This case also highlights the possible co-occurrence of SSD with schizophrenia, presenting a unique clinical profile that underscores the need for personalized treatment approaches. In this case, treatment with a single antipsychotic, 5 mg of olanzapine, effectively reduced the symptoms with the goal of titration on subsequent follow-up. Understanding SSD’s manifestation can provide clinicians with better diagnostic clarity and treatment approaches, including personalized psychological therapy, medication, and support for co-occurring conditions. Additionally, further research into how schizophrenia and substance use may provoke SSD could improve treatment options for individuals facing these co-occurring presentations.
